# Geelong's Thankoffering for Peace

**Published:** 1920-01-03

**Authors:** R. Lewis James

**Affiliations:** *Daily Post* Office, Scarborough.


*To the Editor of* THE HOSPITAL.

### GEELONG'S THANKOFFERING FOR PEACE.

*To the Editor of* THE HOSPITAL.

SIR,—The citizens of Geelong, Victoria, Australia, have decided to rebuild their infirmary and benevolent asylum as a thankoffering for peace. It is estimated that the job will cost £40,000 to £60,000. When I left the city three months ago, after ten years' service editing the *Geelong Advertiser*, the Mayor and members of the Committee expressed to me a hope that the experience of British hospitals could be put at their disposal, so that the new building may be on the most modern lines. It had not been decided whether plans should be obtained by competition; indeed, no step was to be taken for some time pending litigation, which was expected to add £12,000 to the funds, and pending the holding of one of the great galas by which this city of 20,000 people has distinguished itself through the war—a gala which may yield £10,000. The present hospital is two-storeyed, over fifty years old, and very inconvenient. It is on a central site, and the doctors wish to retain that site. A considerable number of citizens would prefer to move the institution to the side of the bay, only half-a-mile distant, or into the country, where ample land could be obtained for a farm, and the patients would enjoy freedom from noise and dust. If the old site is to be used the hospital must be two or three storeyed; on the other sites it could be on the pavilion system. The counsel of hospital committees and surgeons would be greatly appreciated. Copies of plans and annual reports, etc., could be addressed to the Mayor, Councillor Hitchcock, who offered £1,000 to start the subscription list, and is anxious that the new hospital should be a model for Australia.—Yours very truly,

R. LEWIS JAMES.

*Daily Post* Office, Scarborough.

